# N6-Methyladenosine-Related lncRNAs Are Potential Prognostic Biomarkers and Correlated With Tumor Immune Microenvironment in Osteosarcoma

**DOI:** 10.3389/fgene.2021.805607

**Published:** 2022-02-02

**Authors:** Di Zheng, Ling Yu, Zhun Wei, Kezhou Xia, Weichun Guo

**Affiliations:** Department of Orthopedics, Renmin Hospital of Wuhan University, Wuhan, China

**Keywords:** N6-methyladenosine, long non-coding RNA, osteosarcoma, prognostic signature, immune microenvironment

## Abstract

N6-methyladenosine (m6A) and long non-coding RNAs (lncRNAs) play vital roles in the prognostic value and immune microenvironment of malignant tumors. Here, we constructed a m6A-related lncRNA signature in osteosarcoma samples from TCGA dataset and analyzed the association of the signature with tumor immune microenvironment. m6A-related lncRNAs were identified by performing Pearson’s correlation analysis and were used to construct a novel m6A-related lncRNA signature in osteosarcoma. Validation in testing and entire cohorts confirmed the satisfactory accuracy of the risk signature. Principal-component analysis verifies the grouping ability of the risk signature. Functional enrichment analyses connected immune with the risk signature based on the six m6A-related lncRNAs. When patients were separated into high- and low-risk group based on their risk scores, we found that patients in the high-risk group had lower stromal scores, immune scores, and ESTIMATE scores, while the tumor purity was higher in the high-risk group than that in the low-risk group. As for immune cell infiltration, the proportion of monocytes was significantly higher in the low-risk group than that in the high-risk group. Of the six lncRNAs, *AC004812.2* was a protective factor in osteosarcoma and low expression of *AC004812.2* predicted worse overall survival. Overexpression of *AC004812.2* inhibited 143B cell proliferation and increased the expression levels of *IGF2BP1* and *YTHDF1*. In all, our m6A-related lncRNA signature was a potential prognostic biomarker and correlated with tumor immune microenvironment and immune cell infiltration, and *AC004812.2* might be an important regulator of m6A modification and a promising therapeutic target in osteosarcoma.

## Introduction

Osteosarcoma (OS) is the most prevalent primary malignancy of bone that typically affects children and adolescents, with a second peak in incidence in elderly over the age of 50 (Kansara et al., 2014; Tempelaere et al., 2019). In general, osteosarcoma occurs in the metaphysis of long bones and common sites include the femur, tibia, and humerus (Gianferante et al., 2017). Worldwide, osteosarcoma has an incidence of approximately 4–5 cases per million annually (Kansara et al., 2014). Currently, the standard care for osteosarcoma patients was surgical resection combined with neoadjuvant chemotherapy, and adjuvant chemotherapy, which has increased the 5-year survival rate to about 70% for patients with localized disease. Metastasis is the main cause of osteosarcoma-related deaths, and unfortunately osteosarcoma had a property of local invasion and early metastasis (Yang et al., 2020). Despite great success in osteosarcoma management, it had been reported that about half of patients develop lung metastases in the later stage of osteosarcoma and the 5-year survival rate of these patients was less than 30% (Luetke et al., 2014). Moreover, due to intrinsic cellular heterogeneity and the complexity of genetic mechanisms related to the development and progress of osteosarcoma, the prognosis varies even in patients who had similar clinicopathological characteristics and received the same treatments (Zhou et al., 2020; Liu et al., 2021). Thus, it is urgent to identify novel therapeutic target and prognosis biomarkers.

N6-methyladenosine (m6A) is the methylation that occurs at N6-position of adenosine (He et al., 2019). It is a reversible and dynamical RNA epigenetic process that mainly occurs in messenger RNAs (mRNAs) and non-coding RNAs (ncRNAs) that plays a critical role in RNA splicing, stability, export, translation, and other processes (Min et al., 2018; Tang et al., 2018; Berulava et al., 2020). m6A is the most abundant post-transcriptional RNA modification in eukaryotic cells and it is tightly regulated by an expanding list of m6A regulators. According to previous researches, m6A regulators could be divided into three types, including writers, readers, and erasers (Zhao et al., 2017; Huang et al., 2020). The m6A-writer-complex functions as methyltransferase that can install m6A. Then, the m6A modification can be recognized by m6A binding proteins, also known as readers. The erasers are demethylases that are responsible for removing m6A modification. Studies have proved that m6A modifications regulate tumorigenesis and development (Wang et al., 2020). The prognostic value of m6A modifications in cancers is being apparent.

Long non-coding RNAs (lncRNAs) are transcripts that are longer than 200 nucleotides in length but have no protein-coding function (Peng et al., 2017). LncRNAs participate in various aspects of cellular biological processes including cell growth, differentiation, and development. Mechanistically, lncRNAs regulate gene expression at epigenetic, transcriptional, and post-transcriptional levels (Sun et al., 2018). Recent researches had connected aberrant expression of lncRNAs with the development and progress of osteosarcoma. For example, the upregulated lncRNA LINC01123 in osteosarcoma functioned as a competing endogenous RNA *via* sponging miR-516b-5p, resulting in accelerated cell progression (Pan et al., 2021). Besides, lncRNAs could also act as prognostic biomarkers. In osteosarcoma, high expression of lncRNA SNHG4 predicted tumor recurrence and poor overall survival (Xu et al., 2018). Functional study suggested that SNHG4 increased cell viability and invasive potential.

In the present study, we identified m6A-related lncRNAs through performing Pearson’s correlation analysis between the expression profiles of m6A regulators and lncRNAs. We then explored the prognostic ability of these m6A-related lncRNAs and constructed a six m6A-related lncRNA signature to predict the prognosis of osteosarcoma patients. In addition, we detected the association of the prognostic signature with tumor immune environment and immune cell infiltration. Of the six m6A-related lncRNAs, *AC004812.2* was a protective factor and lower expression of *AC004812.2* correlated with worse overall survival in osteosarcoma patients. Overexpression of *AC004812.2* inhibited cell proliferation and increased the expression levels of *IGF2BP1* and *YTHDF1*. In all, our findings provided a promising prognostic indicator of osteosarcoma and suggested that *AC004812.2* might be an important regulator of m6A modification in osteosarcoma.

## Materials and Methods

### Data Collection

The RNA-sequencing data (fragments per kilobase of transcript per million mapped reads value) and clinical information of 88 osteosarcoma samples were downloaded from the TCGA database (https://portal.gdc.cancer.gov/). Patients with missing survival information were excluded from further analysis. LncRNAs and mRNAs were annotated according to the GRCh38 (Genome Reference Consortium Human Build 38) annotation file obtained from GENCODE website.

### Selection of m6A Regulators and m6A-Related lncRNAs

A total of 21 m6A regulators were selected according to previous studies (Tu et al., 2020; Xu et al., 2021), including 11 readers (IGF2BP1, IGF2BP2, IGF2BP3, YTHDC1, YTHDC2, YTHDF1, YTHDF2, YTHDF3, HNRNPC, RBMX, and HNRNPA2B1), 8 writers (METTL3, METTL14, METTL16, VIRMA, RBM15, RBM15B, ZC3H13, and WTAP), and 2 erasers (FTO, ALKBH5). The expression matrixes of the 21 m6A regulators and lncRNAs were extracted. m6A-related lncRNAs were screened by performing Pearson’s correlation analysis between the m6A regulators and lncRNAs based on the expression level. LncRNAs with FDR <0.001 and an absolute Pearson correlation coefficient >0.4 were regarded as m6A-related lncRNAs and were selected for subsequent analysis.

### Construction and Validation of a m6A-Related lncRNA Signature

The TCGA osteosarcoma cohort (entire cohort) was randomly divided into a training cohort and a validation cohort at a ratio of approximately 1:1. The training cohort was utilized to construct the m6A-related lncRNA signature, and the testing cohort and entire cohort were applied to validate the signature. In the training cohort, univariate Cox regression analysis was performed to screen m6A-related prognostic lncRNAs. Thereafter, least absolute shrinkage and selection operator (LASSO) regression analysis was conducted using the *glmnet* package in R software and we found 10 m6A-related lncRNAs were distinctly related to the overall survival of osteosarcoma patients in training cohort. Using the multivariate Cox regression analysis, we ultimately established a risk signature comprising six m6A-related lncRNAs. The risk scores were calculated as follows: risk score = $\sum_{i}^{n}\mathrm{Ex}p_{i}\mathrm{Co}e_{i}$ (Exp = expression level of lncRNAs; Coe = regression coefficient). Patients in the training, testing, and entire cohorts were stratified into high- and low-risk groups according to the median value of risk score in the training cohort. Kaplan–Meier survival analysis was performed to evaluate the prognosis of patients in different subgroups using the *survival* package in R software. Time-dependent ROC analysis was carried out to evaluate the sensitivity and specificity of the risk signature in predicting the prognosis of osteosarcoma patients using the *survivalROC* package.

### Identification of Differentially Expressed Genes and Functional Analysis

Differentially expressed genes (DEGs) between high- and low-risk group were identified using the *limma* package in R software. DEGs were screened out with the criteria of | log2FC |> 1 and FDR <0.05. Gene Ontology (GO) and Kyoto Encyclopedia of Genes and Genomes (KEGG) pathway enrichment analyses were conducted using the *clusterProfiler* package in R software. The results were visualized using the *ggpubr* and *ggplot2* packages. Gene set enrichment analysis (GSEA) were performed to identify pathways that were significantly enriched in high- or low-risk group using the GSEA software (version 4.0.2).

### Tumor Immune Microenvironment and Immune Cell Infiltration

We applied ESTIMATE algorithm to calculate the stroma, immune, and ESTIMATE scores and further predicting tumor purity in TCGA osteosarcoma samples using *estimate* package in R software. The stroma, immune, and ESTIMATE scores and tumor purity between high- and low-risk groups were compared. To explore the degree of immune cell infiltration among the two groups, we utilized CIBERSORT algorithm to calculate the proportion of 22 types of immune cells in TCGA osteosarcoma samples. Samples with *p*-value less than 0.05 in CIBERSORT analysis were selected for subsequent analysis.

### Principal-Component Analysis (PCA)

Principal-component analysis was conducted for effective dimensionality reduction, model identification, and grouping visualization of high-dimensional data of the entire gene expression patterns, 21 m6A regulators, 352 m6A-related lncRNAs, and the six m6A-related lncRNA risk signatures. The results were visualized using *scatterplot3d* package in R software.

### Cell Culture and Transfection

Human osteosarcoma 143B cell line was obtained from the Wuhan Servicebio Technology Co., Ltd. (Wuhan, China). Cells were maintained in α-MEM medium supplied with 10% FBS (fetal bovine serum) and 1% PS (penicillin and streptomycin) under standard culture conditions (37°C, 5% CO_2_). For cell transfection, a total of 3 μg of AC004812.2 overexpression plasmid or empty vector were diluted in 200 μl Opti-MEM (Gibco; Thermo Fisher Scientific, Waltham, MA, United States), and then transfected into 143B cells using Lipofectamine 2000 reagent (Invitrogen, Thermo Fisher Scientific, Waltham, MA, United States) according to the manufacturer’s protocol. The transfection efficiency was examined 48 h after transfection by performing qRT-PCR.

### Cell Proliferation Assay

Cell proliferation was detected using the CCK-8 reagent (Beyotime, Shanghai, China). Briefly, cells transfected with empty vector or AC004812.2 overexpression plasmid were harvested and resuspended with α-MEM medium. Then 5 × 10^3^ cells were added into 96-well plates and the CCK-8 reagent was added into each well at indicated times (0, 24, 48, and 72 h). The absorbance at 450 nm was detected using a microplate reader.

### Quantitative Real-Time (qRT-PCR)

Total RNA was extracted from 143B cells using the TRIzol reagent (Invitrogen, Carlsbad, CA, United States). A total of 1 μg RNA was utilized to synthesize cDNA using the First Strand cDNA Synthesis Kit (Thermo Fisher Scientific, United States). The mRNA expression of indicated genes was quantified using SYBR GREEN (Bio-Rad, CA, United States) and normalized to the expression of housekeeping gene GAPDH. The primer sequences for qRT-PCR are exhibited in Table 1.

**TABLE 1:** The primer sequences of target genes

	—	—
*AC004812.2*	F primer (5′–3′)	TCT​GCG​TGG​TAT​GGT​ACT​CC
	R primer (5′–3′)	GCA​CAT​GAG​CAG​CTC​TTC​TC
*IGF2BP1*	F primer (5′–3′)	GTG​AGC​AAG​TGA​ACA​CCG​AG
	R primer (5′–3′)	CAT​TAT​TTC​CTG​CTC​GGC​CC
*IGF2BP2*	F primer (5′–3′)	GCC​GGA​GCC​TCT​ATC​AAG​AT
	R primer (5′–3′)	TCT​TGC​CAC​CTT​TGC​CAA​TC
*IGF2BP3*	F primer (5′–3′)	CCG​CAG​TTT​GAG​CAA​TCA​GA
	R primer (5′–3′)	TCC​CTG​AGC​CTT​GAA​CTG​AG
*YTHDC1*	F primer (5′–3′)	TGG​TTC​AGG​CAC​AGA​TGG​AT
	R primer (5′–3′)	CCA​TAC​ACC​CTT​CGT​TTG​G
*YTHDC2*	F primer (5′–3′)	TCT​GAG​AAT​TGG​GCT​GTC​GT
	R primer (5′–3′)	GCG​TCA​CTG​CTG​AAC​AAC​AT
*YTHDF1*	F primer (5′–3′)	GCA​ACT​CTC​CTG​GAA​ACG​TC
	R primer (5′–3′)	ACT​CCC​ATT​GAC​GCT​GAA​GA
*YTHDF2*	F primer (5′–3′)	GGA​GTA​GGA​CAG​TCT​CAG​GC
	R primer (5′–3′)	CAC​TGC​CGT​TGA​CAC​TGA​AA
*YTHDF3*	F primer (5′–3′)	ACA​GGC​TGG​ATT​TGG​CAA​TG
	R primer (5′–3′)	TTT​CGG​TTG​AGG​TTT​GGC​AG
*HNRNPA2B1*	F primer (5′–3′)	AGC​TGT​TTG​TTG​GCG​GAA​TT
	R primer (5′–3′)	AGT​TGC​CTC​CTC​TTC​CAC​TC
*HNRNPC*	F primer (5′–3′)	GAC​AGA​TCC​TCG​CTC​CAT​GA
	R primer (5′–3′)	GGT​TCA​CTT​TTG​GCT​CTG​CA
*RBMX*	F primer (5′–3′)	AAG​GAT​GCA​GCC​AGA​GAC​AT
	R primer (5′–3′)	CGT​GGT​GGT​GGT​GCA​TAA​TC
*METTL3*	F primer (5′–3′)	CAC​TGC​TTG​GTT​GGT​GTC​AA
	R primer (5′–3′)	TAC​CTT​TGC​TTG​AAC​CGT​GC
*METTL14*	F primer (5′–3′)	GAT​GAG​ATT​GCA​GCA​CCT​CG
	R primer (5′–3′)	TTT​GAT​CCC​CAT​GAG​GCA​GT
*METTL16*	F primer (5′–3′)	GAC​CTC​CGC​CTA​GTT​CTG​TT
	R primer (5′–3′)	CGC​TTA​CTT​GGT​GGT​GAT​GG
*VIRMA*	F primer (5′–3′)	TCT​GTG​ACT​GTC​TGT​TGG​CT
	R primer (5′–3′)	AGA​TGC​AAG​CTC​TCC​TGT​GT
*RBM15*	F primer (5′–3′)	CGC​GGC​CAG​ACT​AGT​ACT​TA
	R primer (5′–3′)	GCG​TAT​GGT​GCC​AAA​TCG​AT
*RBM15B*	F primer (5′–3′)	CTA​TGC​GGT​CCT​CTT​AGC​CA
	R primer (5′–3′)	GCC​CAA​TGT​CCT​TAG​TGC​TG
*ZC3H13*	F primer (5′–3′)	CAC​ACC​TGG​AGC​TGT​TAT​GC
	R primer (5′–3′)	AGA​ATC​CCG​TCT​GAA​GCA​CA
*WTAP*	F primer (5′–3′)	TAC​CTC​AAG​CAA​GTC​CAG​CA
	R primer (5′–3′)	GTT​GTG​CAA​TAC​GTC​CCT​GG
*FTO*	F primer (5′–3′)	GTG​CTC​AAC​AGG​AAC​CTT​GG
	R primer (5′–3′)	CAG​TTG​AGC​CAT​GGG​TTG​AC
*ALKBH5*	F primer (5′–3′)	GTA​TCA​GGA​GGA​CTC​GGA​CC
	R primer (5′–3′)	AAG​TAC​TTG​TTG​CGC​AGT​GG
*GAPDH*	F primer (5′–3′)	CTG​AGT​ACG​TCG​TGG​AGT​CC
	R primer (5′–3′)	GTC​TTC​TGG​GTG​GCA​GTG​AT

### Statistical Analysis

All the statistical analyses were performed using R software (version 4.1.0) and GraphPad Prism 8.0. Kaplan–Meier survival analysis and the log-rank test were carried out to compare the overall survival between groups. Pearson’s correlation analysis was conducted to analyze the correlation between lncRNAs and mRNAs. Two-tailed Student’s *t*-test was used for comparison of gene expression or scores between two groups. A *p*-value of less than 0.05 was regarded as statistically significant.

## Results

### Identification of m6A-Related lncRNAs in Osteosarcoma

**FIGURE 1:**
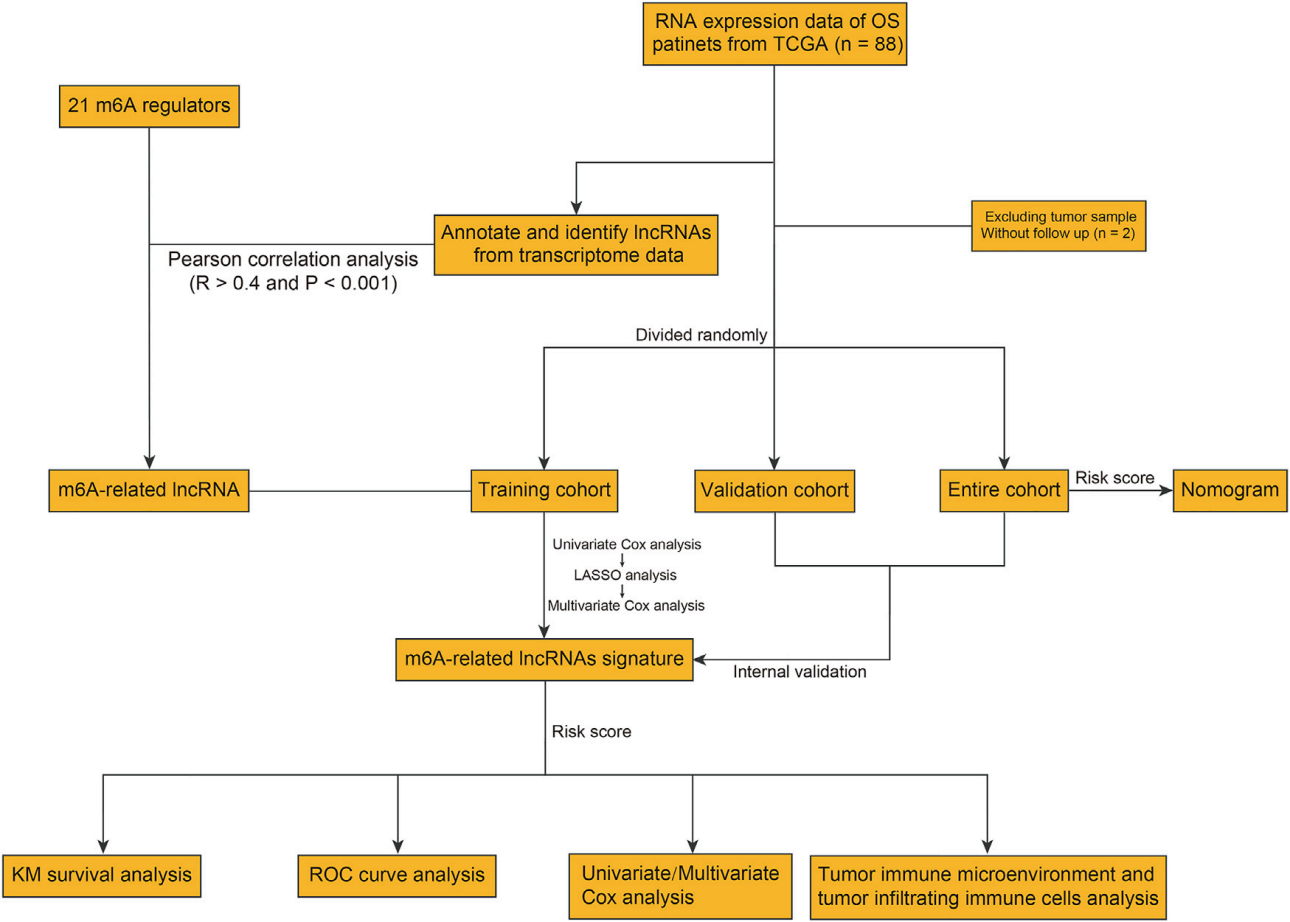
Flowchart of the present study.

**FIGURE 2:**
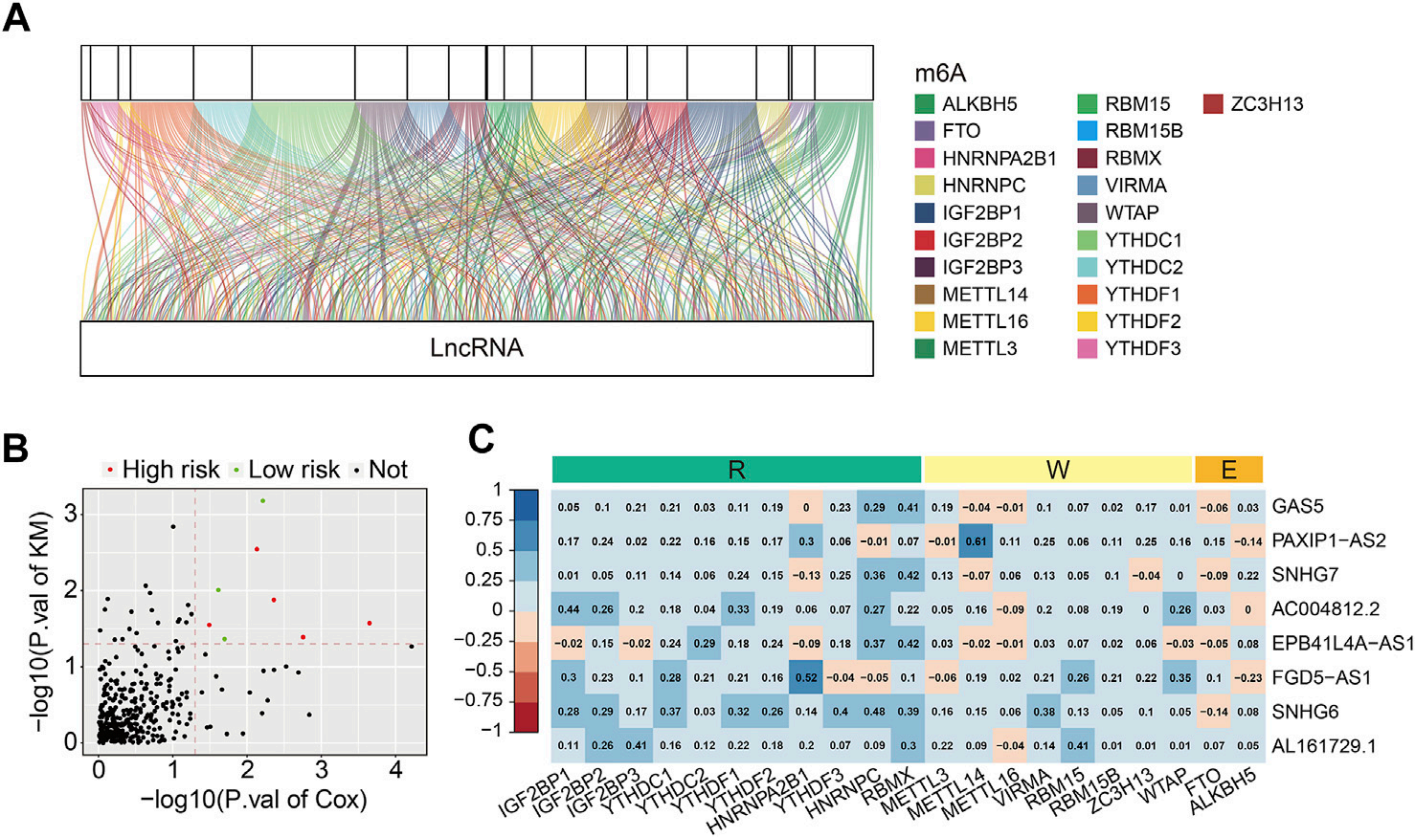
Identification of prognostic m6A-related lncRNAs and correlation analysis of the lncRNAs with m6A-related genes. **(A)** Sankey relational diagram for 21 m6A-related genes and m6A-related lncRNAs. **(B)** Volcano plot exhibited m6A-related prognostic lncRNAs. **(C)** Heatmap for the correlations between the 8 m6A-related prognostic lncRNA and 21 m6A-related genes.

The detailed workflow of the present study is shown in Figure 1. A total of 21 m6A regulators were selected for subsequent investigation according to previous studies. The expression matrixes of the 14,142 lncRNAs and 21 m6A regulators were extracted from the TCGA database. m6A-related lncRNAs were defined as lncRNAs that were significantly correlated with one or more of the 21 m6A regulators (| Pearson R | > 0.4 and FDR <0.001). A total of 352 m6A-related lncRNAs were discerned and the m6A-lncRNA co-expression network is exhibited in Figure 2A. Then, using the univariate Cox regression analysis and Kaplan–Meier survival analysis, we identified 8 robust prognostic lncRNAs among the 352 m6A-related lncRNAs, with 5 lncRNAs (*AL161729.1*, *EPB41L4A-AS1*, *SNHG7*, *GAS5*, and *SNHG6*) were considered as risk factors and 3 lncRNAs (*FGD5-AS1*, *PAXIP1-AS2*, and *AC004812.2*) were protective factors for osteosarcoma patients (Figure 2B). The correlation of the 21 m6A regulators and 8 robust prognostic lncRNAs in the TCGA osteosarcoma entire cohort are shown in Figure 2C.

### Construction of a Risk Signature Based on the m6A-Related lncRNAs in Osteosarcoma

**FIGURE 3:**
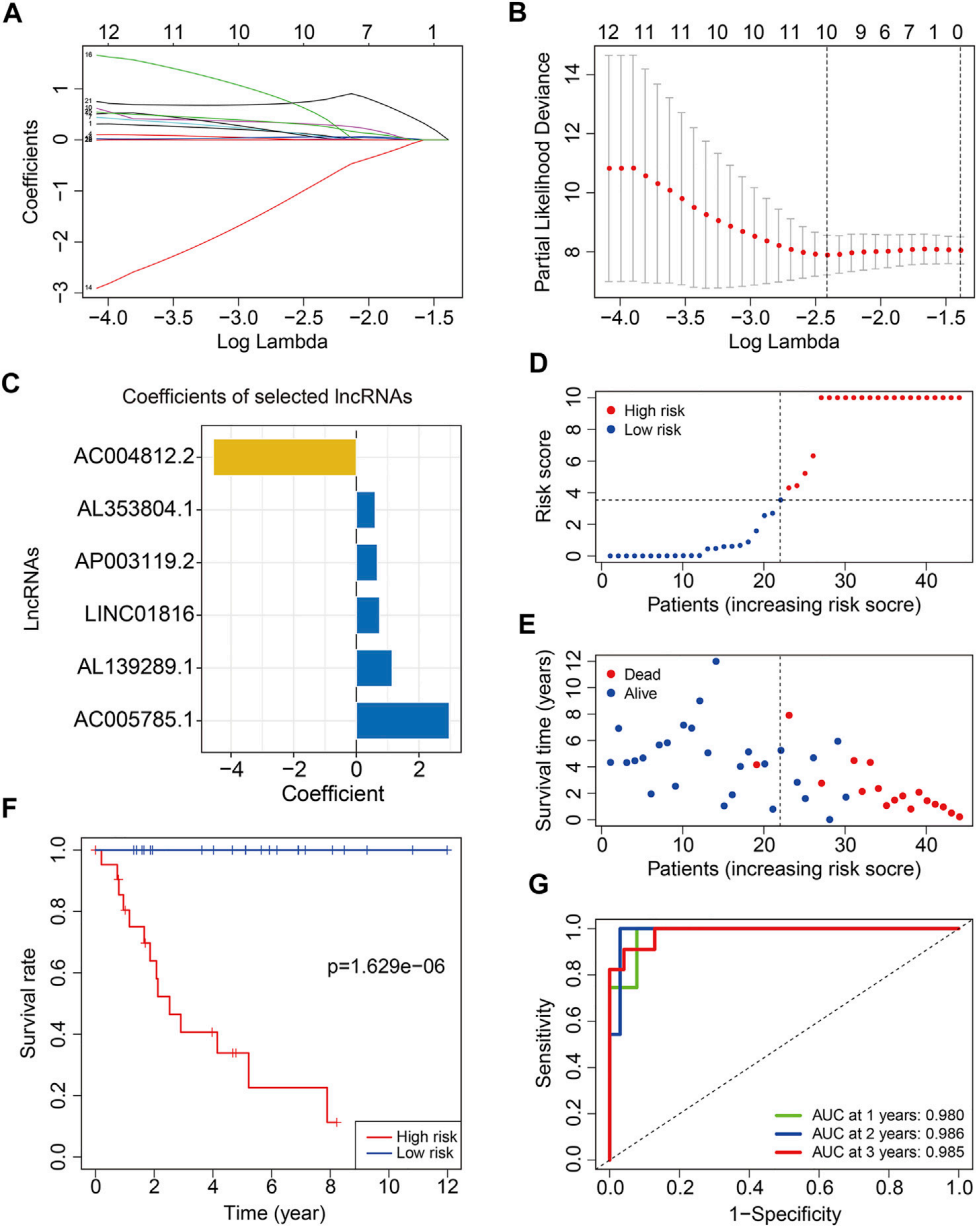
Construction of a six m6A-related lncRNA signature in training cohort. **(A,B)** Least absolute shrinkage and selection operator (LASSO) regression analysis of m6A related lncRNAs. **(C)** Coefficients of the six m6A-related lncRNAs. **(D)** Risk scores distribution based on the six m6A-related lncRNA signature. **(E)** Survival status and survival time of osteosarcoma patients in training cohort. **(F)** Kaplan–Meier survival curves of the overall survival of patients in the high- and low-risk groups. **(G)** ROC curve of the risk signature for predicting 1-year, 2-year, and 3-year survival.

To construct a risk model on the basis of m6A-related lncRNAs in osteosarcoma, we first performed univariate Cox regression analysis to screen out m6A-related prognostic lncRNAs in TCGA training cohort. Then, Lasso regression analysis followed by step-wise multivariate Cox regression analysis were performed and it ultimately generated a m6A-related lncRNA prognostic signature to assess the prognostic risk of patients with osteosarcoma (Figures 3A,B). The signature contained six m6A-related lncRNAs including *AP003119.2*, *LINC01816*, *AL139289.1*, *AC004812.2*, *AC005785.1*, and *AL353804.1*, and their coefficients are displayed in Figure 3C. The risk score was calculated as follows: risk score = *AP003119.2* × 0.6709 + *LINC01816* × 0.7443 + *AL139289.1* × 1.1434 + *AC004812.2* × (−4.5629) + *AC005785.1* × 2.9688 + *AL353804.1* × 0.6036. Subsequently, patients in the training cohort were classified into high- and low-risk groups according to the median value of risk score. The risk score distribution of patients in high- and low-risk groups are depicted in Figure 3D. The survival status and survival time of osteosarcoma patients in the two groups are exhibited in Figure 3E, and it suggested that patients in the high-risk group tended to have higher death rate and shorter survival time. Kaplan–Meier survival analysis demonstrated that the overall survival of the high-risk group was worse than that of the low-risk group (Figure 3F). Time-dependent ROC analysis was utilized to analyze the accuracy of the six m6A-related lncRNA signature in predicting the prognosis of osteosarcoma patients. The area under the curve (AUC) of 1-year, 2-year, and 3-year survival was 0.980, 0.986, and 0.985, respectively (Figure 3G), suggesting that the signature harbored a promising ability to predict the overall survival of osteosarcoma patients.

### Validation of the Six m6A-Related lncRNA Signature in TCGA Testing and Entire Cohorts

**FIGURE 4:**
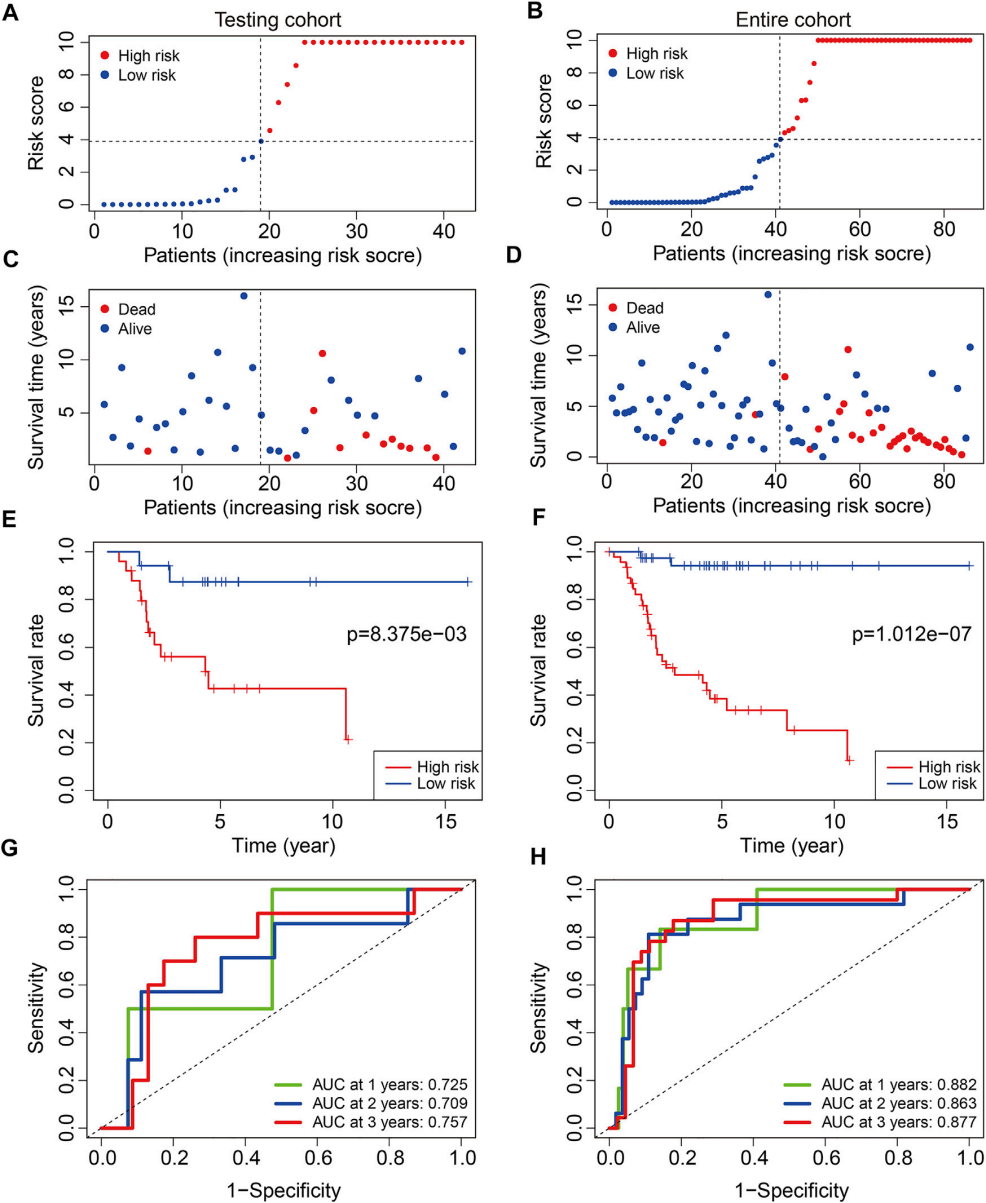
Validation of the six m6A-related lncRNA signature in testing cohort and entire cohort. **(A,B)** Risk scores distribution based on the six m6A-related lncRNA signature in testing cohort and entire cohort. **(C,D)** Survival status and survival time of osteosarcoma patients in testing cohort and entire cohort. **(E,F)** Kaplan–Meier survival curves of the overall survival of osteosarcoma patients in high- and low-risk groups. **(G,H)** ROC curve of the risk signature for predicting 1-year, 2-year, and 3-year survival in testing cohort and entire cohort.

**FIGURE 5:**
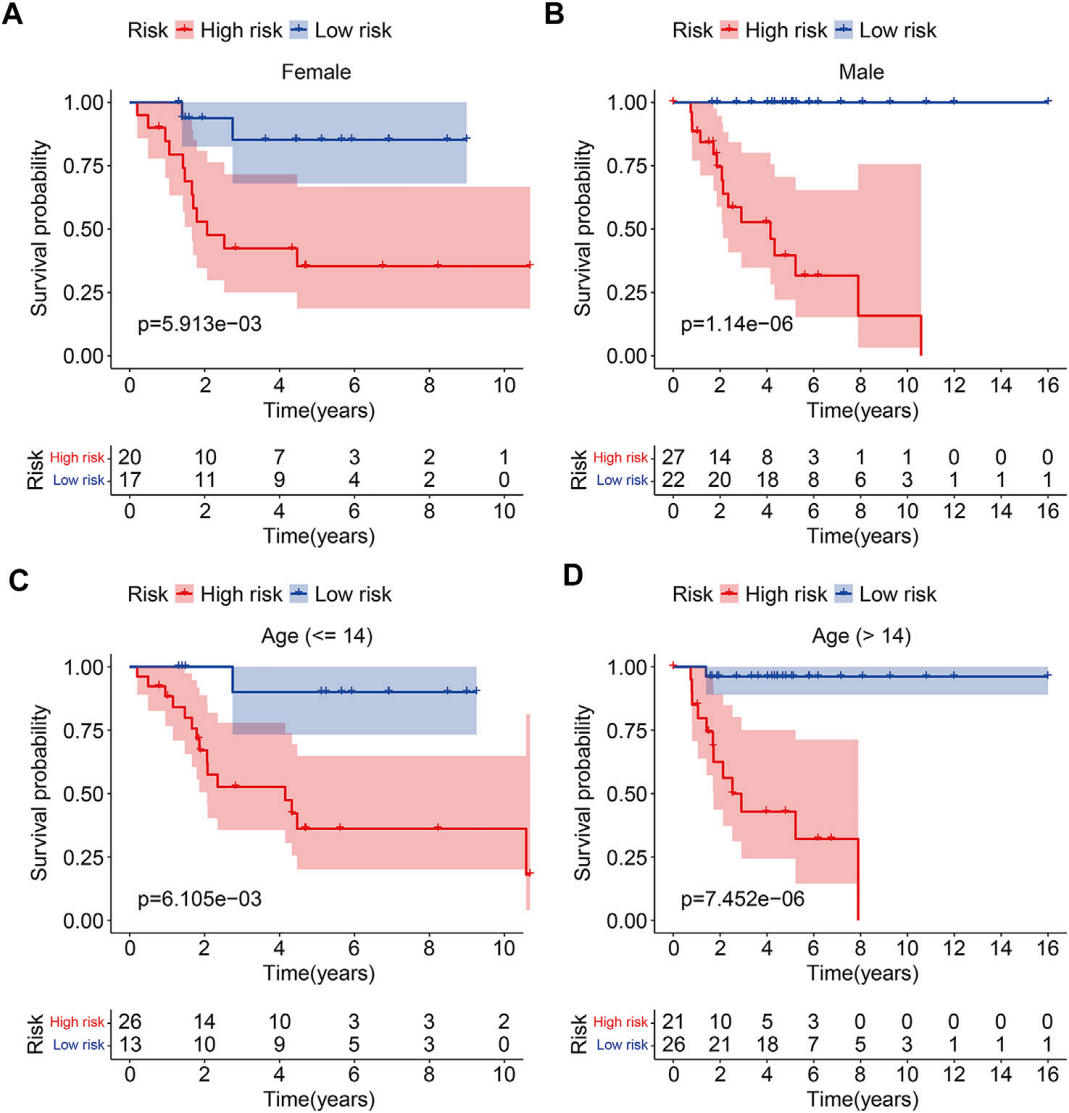
Kaplan–Meier curves of the overall survival differences classified by gender **(A,B)** or age **(C,D)** between the high- and low-risk groups in TCGA osteosarcoma entire cohort.

To test the prognostic capability of the six m6A-related lncRNA signature, we calculated the risk score of each patient in testing cohort and entire cohort using the same formula aforementioned. The risk scores distribution of patients in the two cohorts are exhibited in Figures 4A,B. Figures 4C,D depict the survival status and survival time of patients in the two cohorts. Kaplan–Meier survival analysis was performed in the two cohorts and the results suggested that patients with lower risk score had higher overall survival rate and longer survival time than these of patients with higher risk score (Figures 4E,F). The AUC of 1-year, 2-year, and 3-year survival in testing cohort was 0.725, 0.709, and 0.757, respectively (Figure 4G). As for the entire cohort, it was 0.882, 0.863, and 0.877 (Figure 4H). To further assess the prognostic ability of the six m6A-related lncRNA signature, we analyzed the discrepancies in overall survival stratified by the universal clinical characteristics between the high- and low-risk groups in TCGA osteosarcoma entire cohort. Kaplan–Meier survival analysis indicated that the prognosis of low-risk group continued to be superior to that of high-risk group in the subgroups stratified by gender or age (Figures 5A–D). These results suggested that the six m6A-related lncRNA signature had a robust and stable ability in predicting the prognosis of patients with osteosarcoma.

### Principal-Component Analysis (PCA)

**FIGURE 6:**
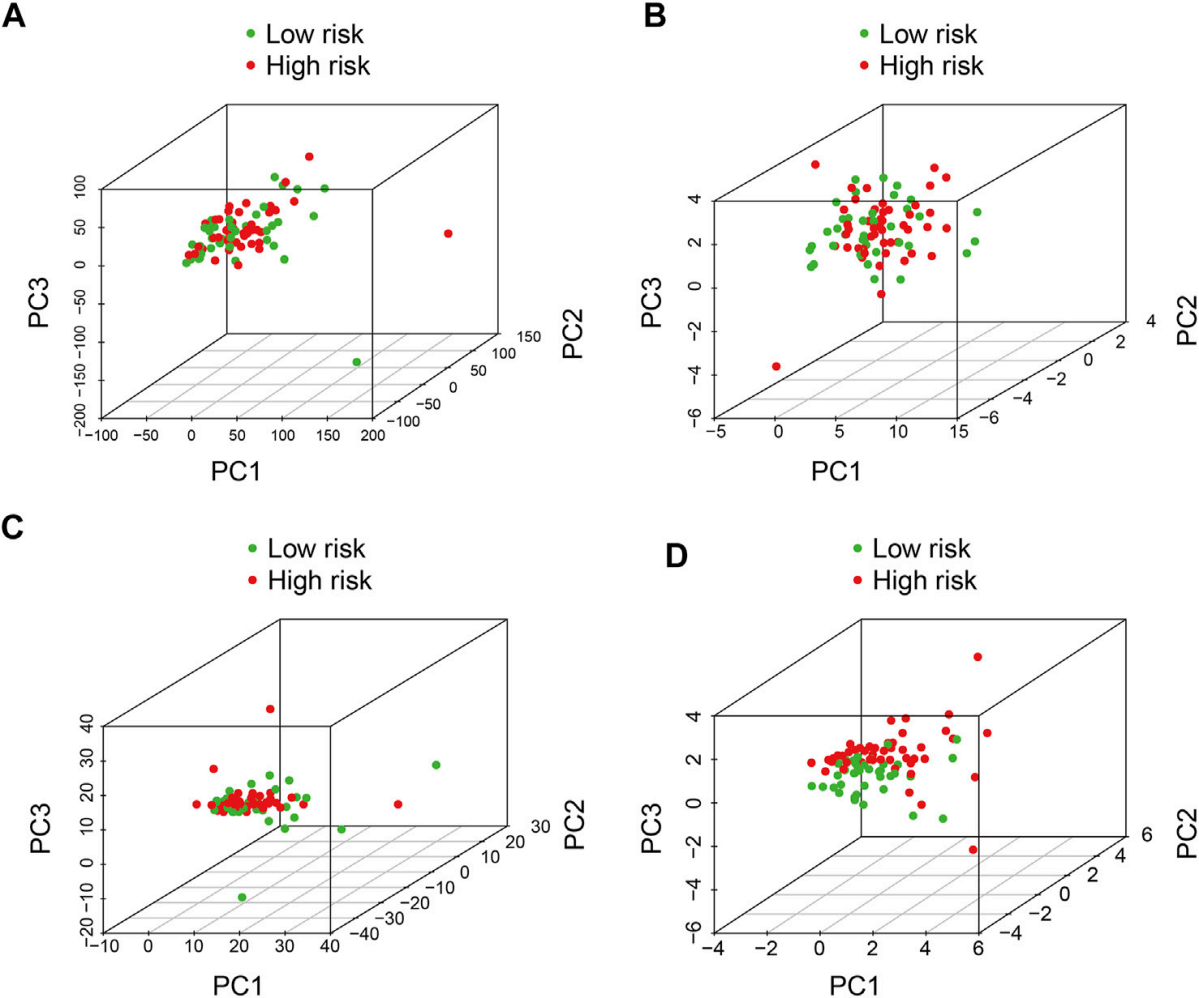
Principal component analysis between the high- and low-risk groups based on **(A)** entire gene expression profiles, **(B)** m6A-related genes, **(C)** m6A-related lncRNAs, and **(D)** risk model based on the representation profiles of the six m6A-related lncRNAs in the TCGA osteosarcoma entire set.

Principal-component analysis was conducted to investigate different distributions between the high- and low-risk groups. The results suggested that the distribution of high- and low-risk groups was relatively scattered based on entire gene expression patterns (Figure 6A), 21 m6A regulators (Figure 6B), and 352 m6A-related lncRNAs (Figure 6C). However, the result obtained on the basis of the risk signature displayed that high- and low-risk group tended to have different distributions (Figure 6D), indicating the six m6A-related lncRNA signature could distinguish between high- and low-risk group.

### The Prognostic Independence of the Six m6A-Related lncRNA Signature and Construction of a Nomogram in Osteosarcoma

To evaluate whether the six m6A-related lncRNA signature was an independent prognostic factor for osteosarcoma patients, we conducted univariate and multivariate Cox regression analyses in the entire TCGA osteosarcoma cohort. The results illustrated that risk score was the only independent predictor of overall survival in osteosarcoma patients (Table 2).

**TABLE 2:** Univariable and multivariable analyses of the six m6A-related lncRNA signature in TCGA osteosarcoma cohort

Variables	Univariable analysis				Multivariable analysis			
	HR	95% CI of HR		*P*	HR	95% CI of HR		*P*
		Lower	Upper			Lower	Upper	
Gender (female vs. male)	0.6740093	0.3205156	1.4173679	0.2982258	0.7092606	0.3019924	1.6657720	0.4303542
Age (≤14 vs. >14)	0.7192754	0.3418771	1.5132837	0.3852343	0.7862366	0.3334664	1.8537641	0.5826210
Risk score	1.0000755	1.0000408	1.0001102	0.0000198	1.0000787	1.0000419	1.0001154	0.0000273

**FIGURE 7:**
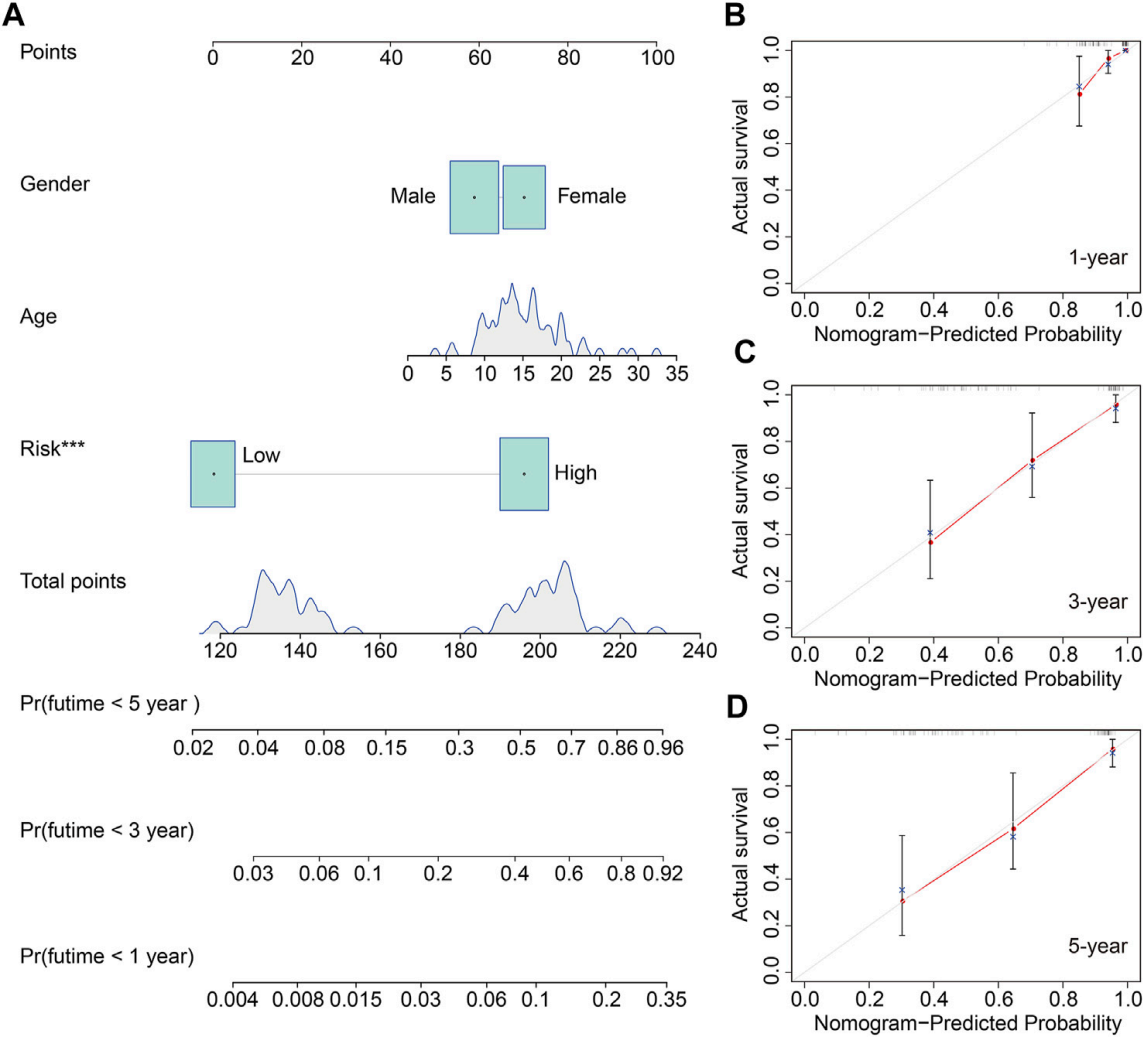
Construction of a nomogram to predict the prognosis of osteosarcoma patients. **(A)** Nomogram based on gender, age, and risk score. **(B–D)** Calibration plots to assess the accuracy of the nomogram in predicting the probability of 1-year, 3-year, and 5-year overall survival of osteosarcoma patients.

We also construct a nomogram for clinical application in predicting the overall survival of patients with osteosarcoma to help in the decision-making for clinicians. The nomogram comprised risk score and clinical characteristic including gender and age. Of these factors, the risk score of the six m6A-related lncRNA signature harbored predominant predictive ability in the nomogram (Figure 7A). The calibration curve illustrated that the predicted rates of 1-year, 3-year, and 5-year overall survival were consistent with the observed overall survival rates at 1 year, 3 years, and 5 years (Figures 7B–D).

### Identification of Risk-Related Differentially Expressed Genes and Functional Analysis

**FIGURE 8:**
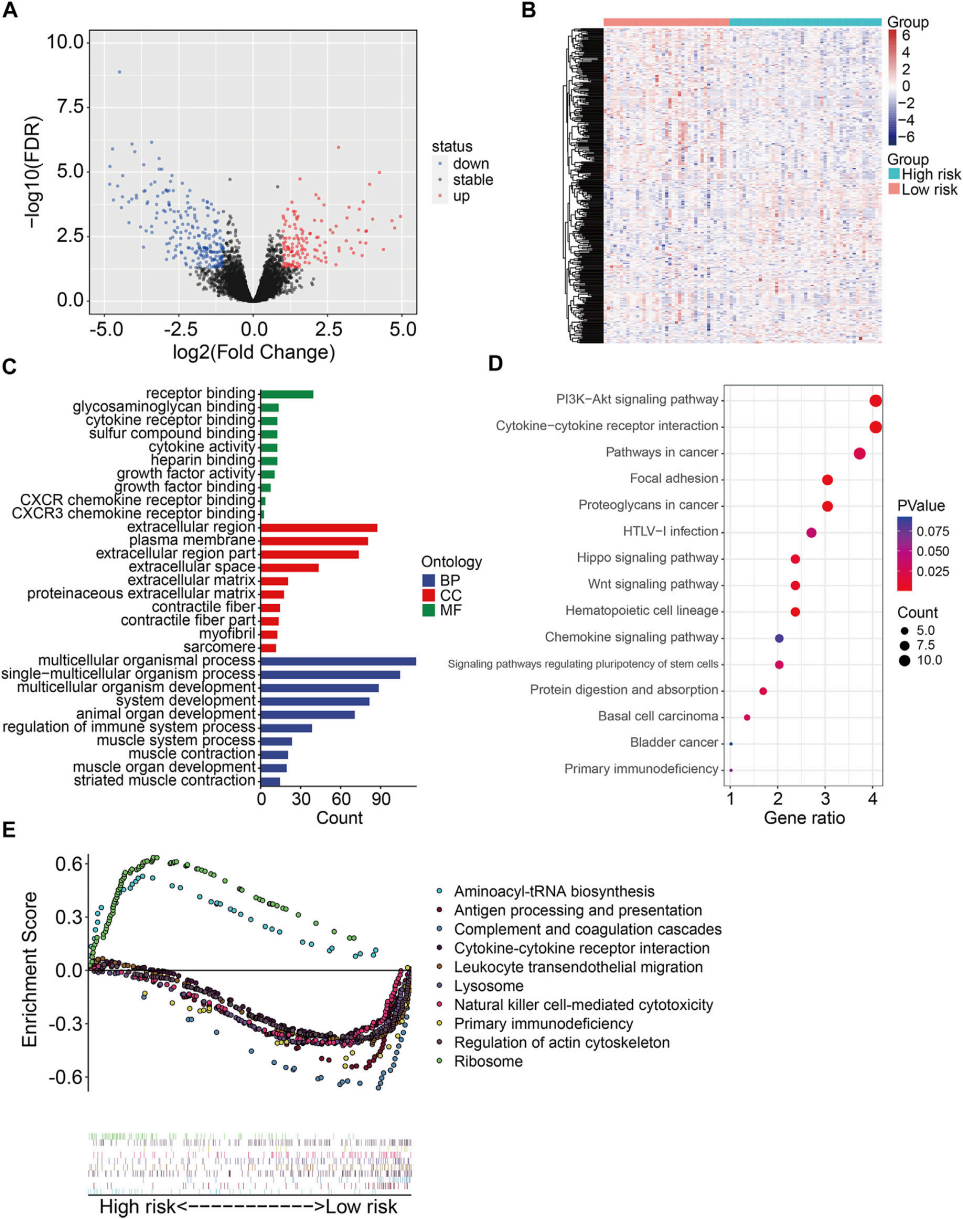
Identification of risk-related differentially expressed genes and functional enrichment analysis. **(A,B)** Volcano plot and heatmap exhibited differentially expressed genes between high- and low-risk groups. **(C)** GO enrichment analysis. **(D)** KEGG enrichment analysis. **(E)** Gene set enrichment analysis.

To further explore the underlying molecular mechanism of the six m6A-related lncRNA signature, we identified differentially expressed genes between high- and low-risk groups in the TCGA osteosarcoma gene expression matrix. A total of 369 DEGs were screened out with the criteria of | log_2_FC | > 1 and FDR <0.05. Of the 369 DEGs, 136 genes were upregulated in high-risk group compared with low-risk group, while 233 genes were downregulated (Figures 8A,B). Then, we performed GO and KEGG enrichment analyses on the DEGs. We found that these DEGs were remarkably enriched in biological processes including multicellular organismal process, single-multicellular organism process, multicellular organism development, system development, and animal organ development (Figure 8C). In terms of molecular function, receptor binding, glycosaminoglycan binding, cytokine receptor binding, sulfur compound binding, and cytokine activity were significantly enriched. As for the cellular component, the DEGs were significantly enriched in the extracellular region, plasma membrane, and extracellular region. KEGG enrichment analysis indicated that PI3K-AKT signaling pathway, Cytokine–cytokine receptor interaction, Pathways in cancer, Focal adhesion, Hippo signaling pathway, and WNT signaling pathway were significantly enriched (Figure 8D). We also performed gene set enrichment analysis in the TCGA osteosarcoma gene expression matrix, and the results revealed that immune-related pathways including antigen processing and presentation, complement and coagulation cascades, cytokine–cytokine receptor interaction, leukocyte trans-endothelial migration, and natural killer cell–mediated cytotoxicity were significantly enriched (Figure 8E). These analyses might give us some insights into the cellular biological effects, especially immune, related to the risk signature based on the six m6A-related lncRNAs.

### Estimation of Tumor Immune Microenvironment and Tumor-Infiltrating Immune Cells Using the Six m6A-Related lncRNA Signature

**FIGURE 9:**
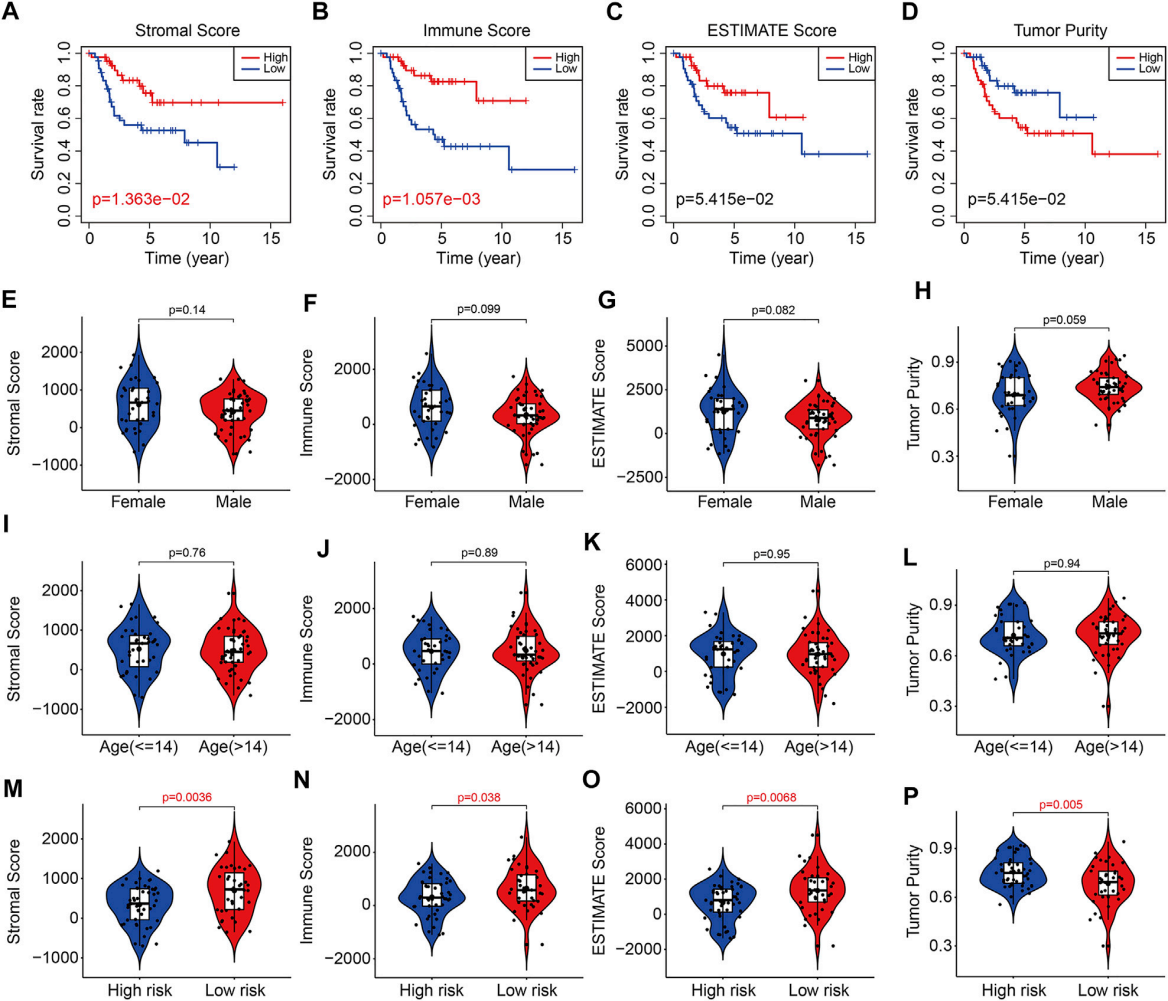
The six-m6A related lncRNA signature correlated with tumor immune microenvironment. **(A–D)** Kaplan–Meier survival curves of high or low stromal scores, immune scores, ESTIMATE scores, and tumor purity. **(E–H)** The stromal scores, immune scores, ESTIMATE scores, and tumor purity in osteosarcoma patients with different gender (female and male). **(I–L)** The stromal scores, immune scores, ESTIMATE scores, and tumor purity in osteosarcoma patients classified by age (≤14 and >14 years). **(M–P)** The stromal scores, immune scores, ESTIMATE scores, and tumor purity in osteosarcoma patients with high and low risk.

With the use of ESTIMATE algorithm, the stromal, immune, and ESTIMATE scores and tumor purity for all TCGA osteosarcoma samples were calculated. As shown in Figures 9A,B, Kaplan–Meier survival analysis suggested that lower stromal scores or immune scores predicted worse prognosis in osteosarcoma. Although the differences were not statistically significant, osteosarcoma patients with lower ESTIMATE scores or higher tumor purity tended to have shorter survival time compared with those with higher ESTIMATE scores or lower tumor purity (Figures 9C,D). Therefore, these analyses indicated that the tumor immune microenvironment was closely associated with the prognosis in osteosarcoma. We then categorized the stromal scores, immune scores, ESTIMATE scores, and tumor purity by risk score and clinical characteristic including gender and age. As shown in Figures 9E–L, the stromal scores, immune scores, ESTIMATE scores, and tumor purity showed no differences between subgroups including female and male, ≤14 years and >14 years. However, the stromal scores, immune scores, and ESTIMATE scores were lower in the high-risk group than those in the low-risk group, and tumor purity was higher in the high-risk group than that in the low-risk group (Figures 9M–P).

**FIGURE 10:**
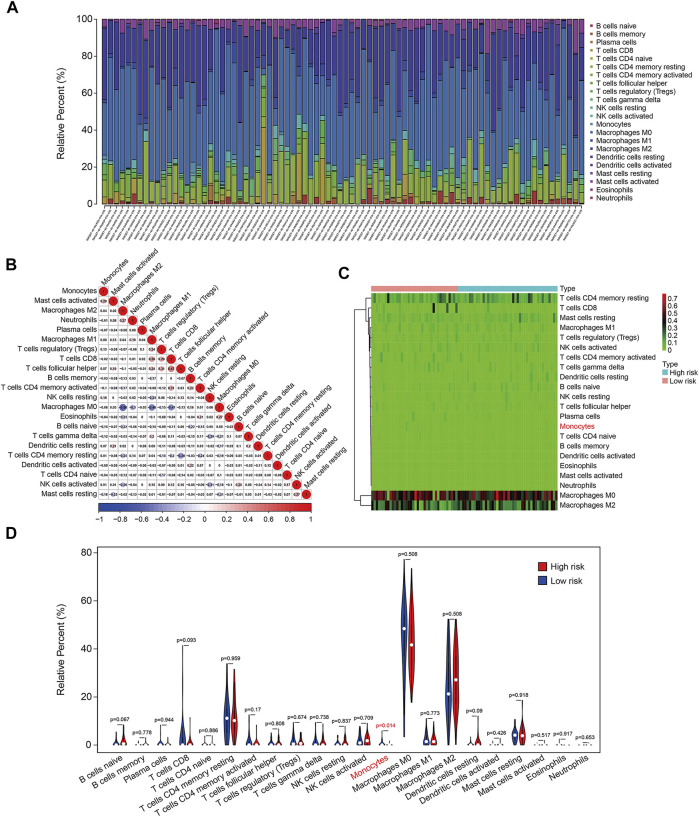
The six-m6A related lncRNA signature correlated with tumor-infiltrating immune cells. **(A)** The proportion of 22 immune cell types in osteosarcoma samples. **(B)** Correlation of the 22 immune cell types. **(C,D)** Heatmap and violin plot visualizing the infiltrated immune cells in high- and low-risk groups.

The infiltration of 22 immune cell types in osteosarcoma samples were further analyzed using the CIBERSORT algorithm. The results revealed that Macrophages M0, Macrophages M2, T cells CD4 memory resting, Mast cells resting, and Macrophages M1 accounted for a large proportion of immune cell infiltration (Figure 10A). The correlation coefficients between 22 types of immune cell are depicted in Figure 10B. Although there were no significant differences in the proportion of most of the infiltrated immune cell types between high- and low-risk group, we found that the proportion of monocyte was significantly higher in the low-risk group than that in the high-risk group (Figures 10C,D). Therefore, we could speculate that monocyte might play a critical role in the development and progress of osteosarcoma.

### Expression and Prognosis Analyses of the Six m6A-Related lncRNAs

**FIGURE 11:**
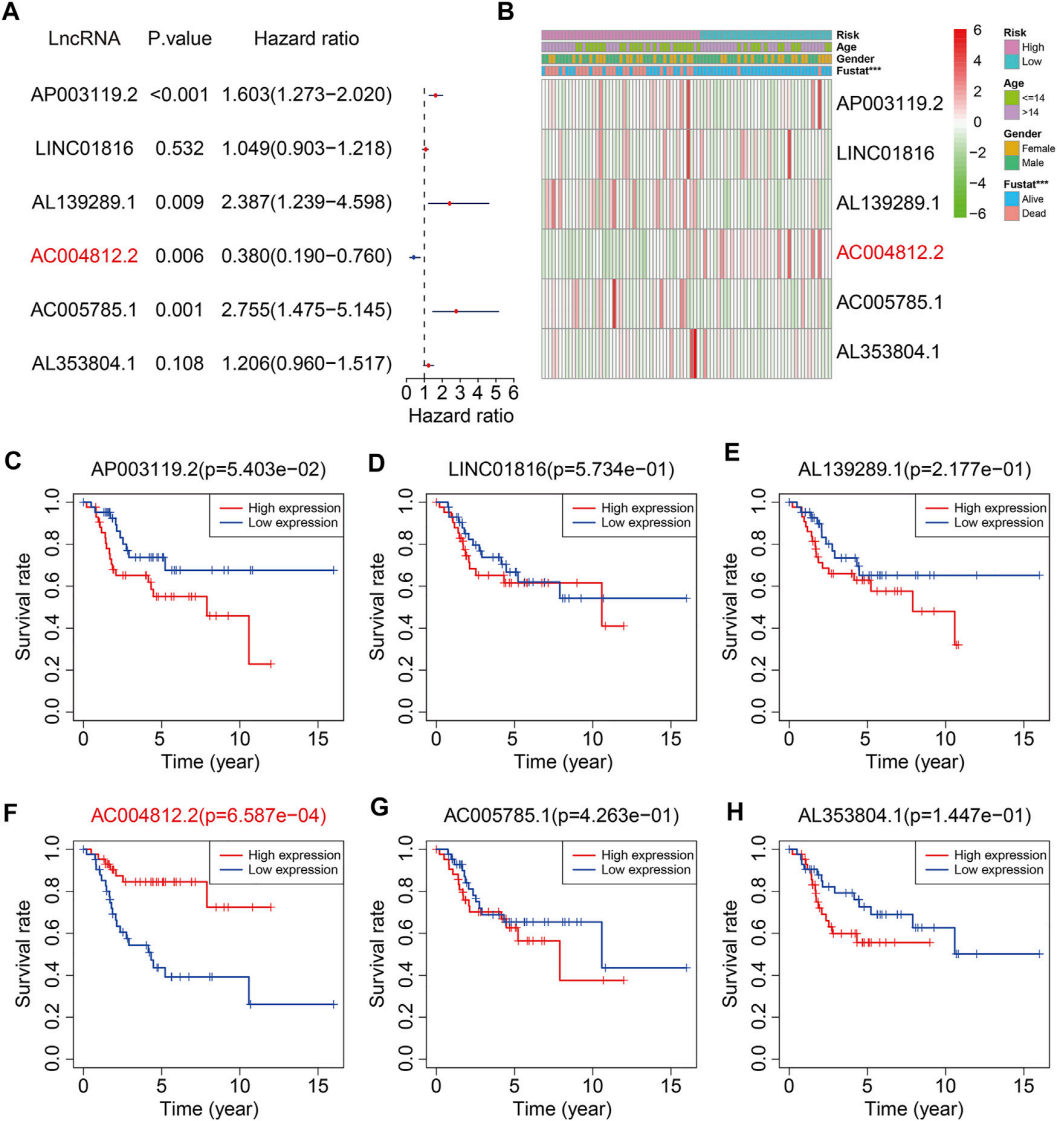
The prognosis predictive value and expression analysis of the six m6A-related lncRNAs. **(A)** Univariate Cox regression analysis of the six m6A-related lncRNAs. **(B)** Heatmap exhibited the expression of the six lncRNAs. **(C–H)** Kaplan–Meier survival analysis of *AP003119.2*, *LINC01816*, *AL139289.1*, *AC004812.2*, *AC005785.1*, and *AL353804.1* in TCGA osteosarcoma cohort.

**FIGURE 12:**
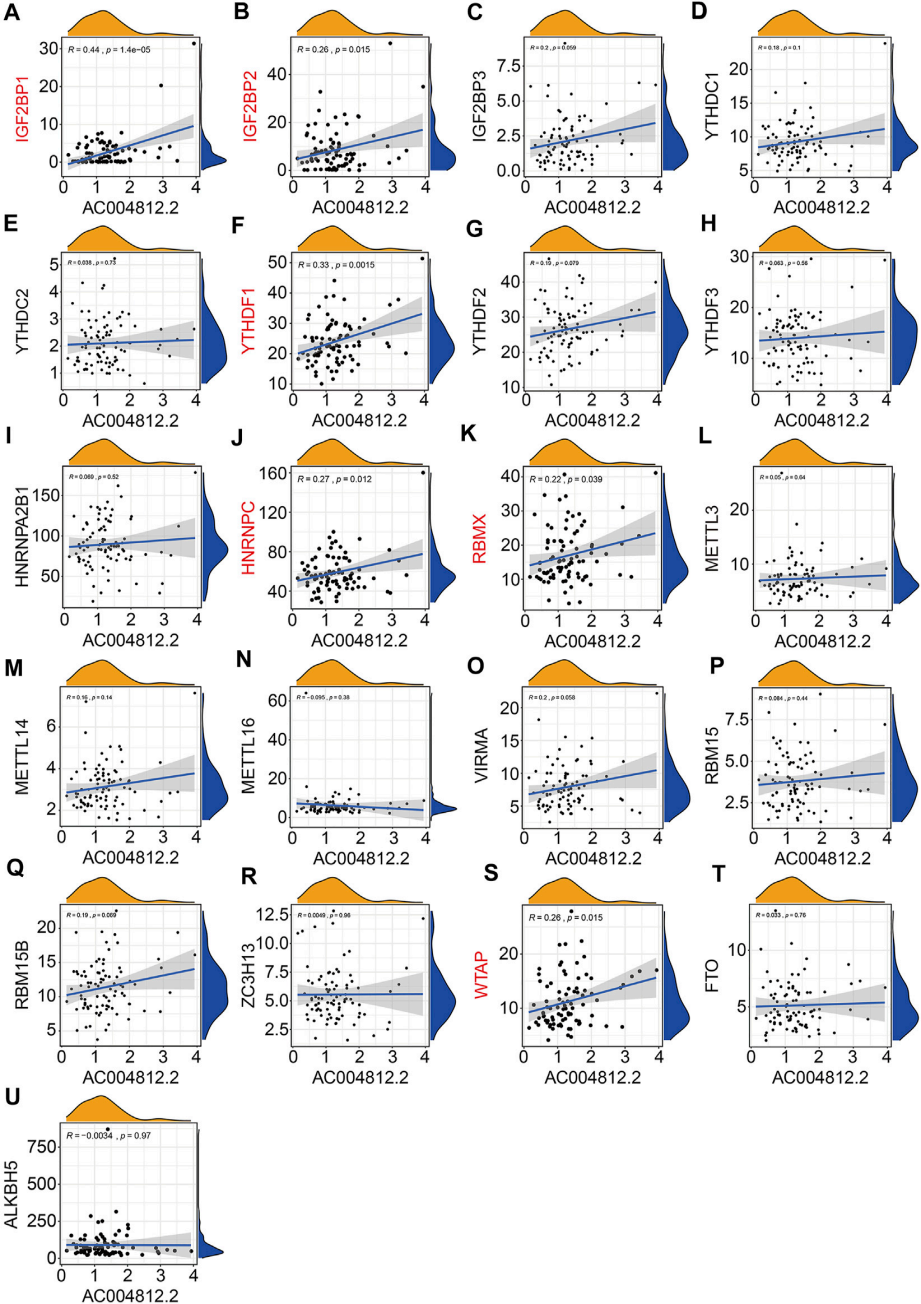
Pearson correlation analysis of *AC004812.2* with m6A-related genes including *IGF2BP1* **(A)**, *IGF2BP2* **(B)**, *IGF2BP3* **(C)**, *YTHDC1* **(D)**, *YTHDC2* **(E)**, *YTHDF1* **(F)**, *YTHDF2* **(G)**, *YTHDF3* **(H)**, *HNRNPA2B1* **(I)**, *HNRNPC* **(J)**, *RBMX* **(K)**, *METTL3* **(L)**, *METTL14* **(M)**, *METTL16* **(N)**, *VIRMA* **(O)**, *RBM15* **(P)**, *RBM15B* **(Q)**, *ZC3H13* **(R)**, *WTAP* **(S)**, *FTO* **(T)**, and *ALKBH5* **(U)**.

Of the six m6A-related lncRNAs, univariate Cox regression analysis indicated that *AP003119.2*, *AL139289.1*, *AC005785.1*, and *AL353804.1* were risk factors (HR > 1, *p* < 0.05), while *AC004812.2* was the only protective factor (HR < 1, *p* < 0.05) in osteosarcoma patients (Figure 11A). Expression analysis suggested that the expression levels of AP003119.2, LINC01816, AL139289.1, AC005785.1, and AL353804.1 were higher in the high-risk group than those in the low-risk group, and conversely, the expression of AC004812.2 was lower in the high-risk group (Figure 11B). Furthermore, we conducted Kaplan–Meier survival analysis on the six lncRNAs and the results suggested that the lower expression of *AC004812.2* significantly correlated with worse overall survival, while the expression of the other five lncRNAs did not relate to the prognosis of osteosarcoma patients (Figures 11C–H). Taken together, these analyses indicated that *AC004812.2* might be a promising prognostic biomarker and therapeutic target in osteosarcoma. We also explore the correlation of *AC004812.2* with the 21 m6A regulators by performing Pearson’s correlation analysis. The results suggested that *AC004812.2* expression was positively correlated with the expression of *IGF2BP1* and *YTHDF1* (| Pearson R | > 0.3, *p* < 0.05) (Figures 12A–U).

### The Effect of *AC004812.2* Overexpression on Cell Proliferation and the Expression of m6A Regulators

**FIGURE 13:**
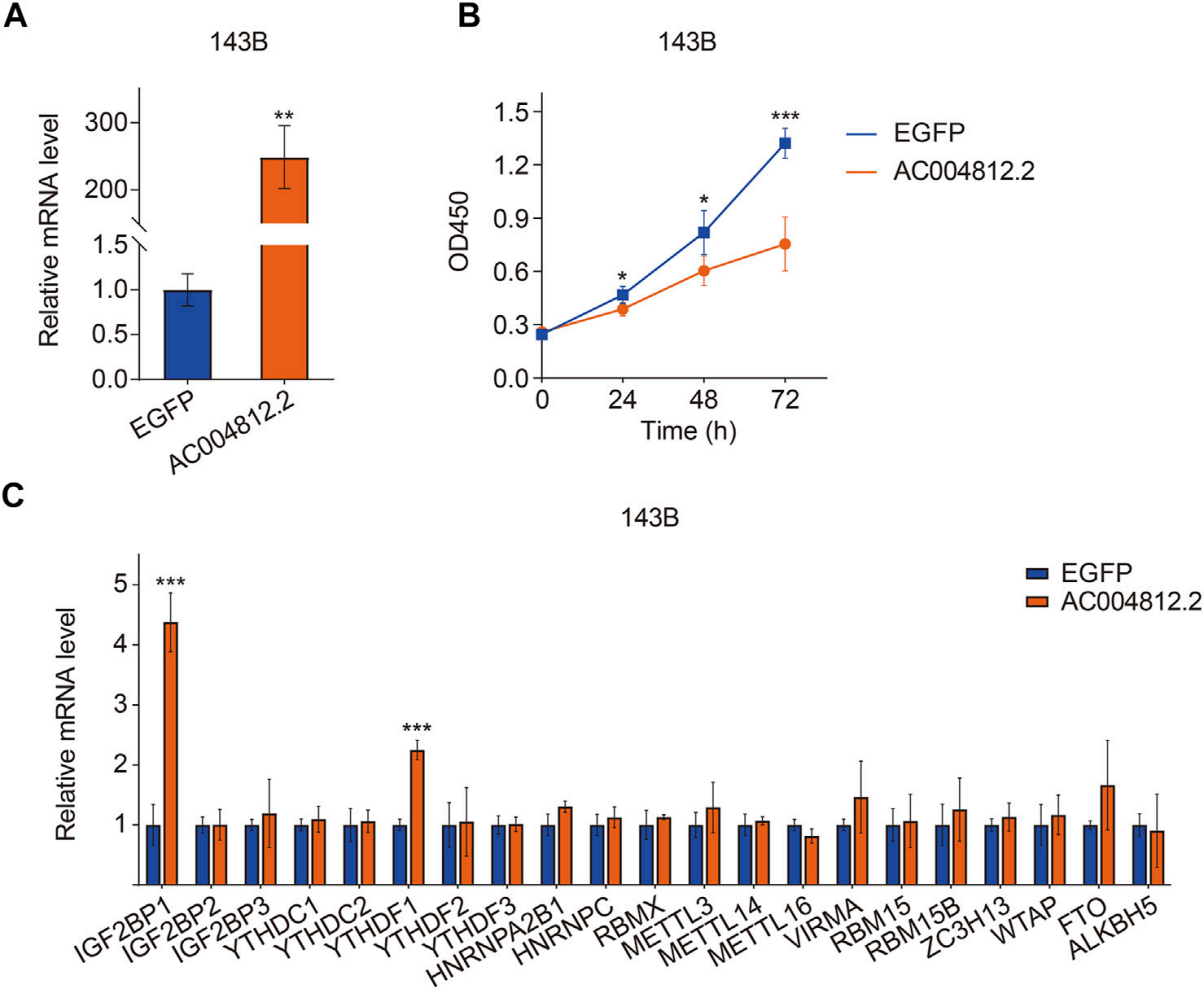
The effect of *AC004812.2* on cell proliferation and the expression of m6A regulators. **(A)** qRT-PCR detected the expression level of *AC004812.2* in 143B cells. **(B)** CCK-8 assay was performed to evaluate the effect of *AC004812.2* on cell proliferation. **(C)** qRT-PCR detected the expression levels of m6A regulators in 143B cells transfected with *AC004812.2* overexpression plasmid. ^**^ *p* < 0.01, ^***^ *p* < 0.001.

To explore the effect of *AC004812.2* on osteosarcoma cell proliferation, we overexpressed *AC004812.2* in 143B cells through plasmid transfection and performed CCK-8 assay. The transfection efficiency was confirmed by qRT-PCR (Figure 13A). The results suggested that overexpression of *AC004812.2* remarkably inhibited cell proliferation (Figure 13B). Then, we conducted qRT-PCR to detect the expression of m6A regulators in 143B cells transfected with *AC004812.2* overexpression plasmid. As shown in Figure 13C, overexpression of *AC004812.2* significantly increased the expression levels of *IGF2BP1* and *YTHDF1*.

## Discussion

With the development of high-throughput sequencing technology and its popularization in cancer researches, revealing the genetic or epigenetic abnormalities during tumor occurrence and development provides an avenue for the identification of therapeutic targets and prognostic biomarkers in various types of cancers (Zhao et al., 2020). Recently, integrated analysis of the high-throughput sequencing results from public databases had drawn attention in numerous cancer fields and led to many important discoveries involved in the treatment and prognosis of tumors (Senft et al., 2017; Shih et al., 2020). Here, we identified 352 m6A-related lncRNAs by performing Pearson’s correlation analysis on the transcriptome data of osteosarcoma samples in TCGA database. We then used them to construct a risk signature for predicting the prognosis of osteosarcoma patients. The risk signature stratified patients into high- and low-risk groups according to the median value of risk score. Kaplan–Meier survival analysis demonstrated that the clinical outcome of patients in low-risk group was better than that in high-risk group. Moreover, when patients were separated into different subgroups by gender or age, the prognosis of low-risk group continued to be superior to that of high-risk group. Besides, the six-m6A related lncRNA signature was an independent prognostic marker in osteosarcoma. In addition, we construct a nomogram comprising the risk signature, age, and gender to help in the decision-making for clinicians. In all, these analyses confirmed the satisfactory accuracy and clinical applicability of the risk signature in predicting the prognosis of patients with osteosarcoma.

To reveal the underlying molecular mechanism of the m6A-related lncRNA signature, we screened out differentially expressed genes between high- and low-risk groups. Functional enrichment analysis indicated that the 369 DEGs were significantly enriched in pathways including PI3K-AKT signaling pathway, Cytokine–cytokine receptor interaction, Pathways in cancer, Focal adhesion, Hippo signaling pathway, and WNT signaling pathway. Further, GSEA suggested that immune-related pathways including antigen processing and presentation, complement and coagulation cascades, cytokine–cytokine receptor interaction, leukocyte trans-endothelial migration, and natural killer cell–mediated cytotoxicity were significantly enriched, indicating that immune status might be correlated with prognosis of osteosarcoma patients. Recently, tumor immune microenvironment (TME) had drawn much attention due to its essential role in cancer initiation and progression (Hinshaw and Shevde, 2019; Lei X. et al., 2020). The TME was mainly constituted by cellular components such as extracellular matrix and signaling molecules, and cellular components including tumor cell, tumor-infiltrating immune cells, endothelial cells, and fibroblasts (Cassim and Pouyssegur, 2019). In osteosarcoma, TME also comprised bone cells. The high heterogeneity and complexity of TME had different effects during cancer progression and was an obstacle in treating cancer (Duan et al., 2020). Targeting the TME might be helpful in removing the obstruction to anticancer immune responses and improving the clinical outcome of immunotherapy (Pitt et al., 2016; Lei Y. et al., 2020). To estimate the TME in osteosarcoma, we used ESTIMATE algorithm to quantify the score of immune cells and stromal cells by analyzing the gene expression data of osteosarcoma samples. We found that lower stromal scores or immune scores predicted worse prognosis in osteosarcoma and patients with lower ESTIMATE scores or higher tumor purity tended to have shorter survival time compared with those with higher ESTIMATE scores or lower tumor purity, suggesting that the TME was a prognostic biomarker in osteosarcoma. We further analyzed the discrepancies in stromal scores, immune scores, ESTIMATE scores, and tumor purity stratified by the universal clinical characteristics and the risk signature. Results showed that the stromal score, immune score, and ESTIMATE scores and tumor purity showed no differences between subgroups including female and male, ≤14 years and >14 years, whereas the stromal, immune, and ESTIMATE scores were lower in the high-risk group than those in the low-risk group, and the tumor purity was higher in the high-risk group than that in the low-risk group. Taken together, these analyses connected the risk signature with the TME. Consistent with the former researches (Hu et al., 2020; Zhang et al., 2020), we found that osteosarcoma was an immunosensitive tumor type that was mainly infiltrated macrophages, T lymphocytes, B lymphocytes, dendritic cells, and mast cells. Although the proportion of monocytes was a little small in infiltrated immune cells, a significant difference of infiltrated monocytes cells was found between high- and low-risk groups, and of which was lower in the high-risk group. In osteosarcoma, patrolling monocytes could inhibit distant metastasis of osteosarcoma and a higher proportion of monocytes predicted a better prognosis (Chen and Zhao, 2020). Thus, infiltration of monocytes has a beneficial impact on the prognosis of osteosarcoma patients and it is well worth investigating the role of infiltrated monocytes on the oncogenesis and progression of osteosarcoma in the future.

m6A RNA methylation, one of the most common modifications in mRNA and ncRNA, was closely related with oncogenesis and tumor development. m6A regulators were potent prognostic biomarkers in various tumors and could regulate tumor cell proliferation, migration, invasion, and metastasis in a m6A-dependent manner (Chen et al., 2019; Li et al., 2019; Chen et al., 2020). In osteosarcoma, the total methylated RNA (m6A) level was increased in osteosarcoma tumor samples than corresponding normal tissues, suggesting a potential role of m6A modification in osteosarcoma occurrence and progression. Aberrant expression of m6A methyltransferase METTL3 increased m6A methylation and total mRNA level of LEF1, resulting in the activation of Wnt/β-catenin signaling pathway and accelerated tumor progression (Miao et al., 2019). In addition, the prognostic value of m6A regulators in osteosarcoma had also been reported. In a tissue microarray cohort, Li et al. (2020) found that low expression of m6A regulators including METTL3, METTL14, and YTHDF2 predicted poor prognosis of osteosarcoma patients, while low expression of KIAA1429 and HNRNPA2B1 correlated with longer overall survival time. The correlation between lncRNAs with m6A modification is complex (Ma et al., 2019). On the one hand, specific lncRNAs could be modified by m6A regulators, resulting in aberrant expression or dysfunction of lncRNAs (Chen et al., 2020). On the other hand, lncRNAs might act as competitive endogenous RNAs to target m6A regulators thus influencing m6A modification level (Wang et al., 2019; Ye et al., 2021). Up to now, little is known about the dynamic regulation of m6A modification and lncRNAs during osteosarcoma progression and its clinical value. Here, we found that of the six m6A-related lncRNAs, *AC004812.2* was a protective factor and lower expression of which predicted poor prognosis in osteosarcoma. Upregulating the expression level of *AC004812.2* remarkably inhibited cell proliferation, further indicating the tumor suppressive role of *AC004812.2*. Moreover, the expression of *AC004812.2* was positively correlated with the two m6A modification readers, *IGF2BP1* and *YTHDF1*. Overexpression of *AC004812.2* increased the mRNA levels of *IGF2BP1* and *YTHDF1*. Thus, it is reasonable to speculate that *AC004812.2* regulates osteosarcoma cell proliferation in a m6A-dependent manner.

Despite the findings aforementioned, there are some innate limitations in our research and need to be addressed. First, more and more m6A regulators are being discovered, and here only the reported m6A regulators were enrolled. Second, due to the lack of external lncRNA expression matrixes and corresponding clinical information, our risk model was not validated in an independent cohort. Third, the relationship between *AC004812.2* and m6A regulators should be further studied and the detailed mechanism of *AC004812.2* in regulating the proliferation of osteosarcoma cells needs to be investigated.

In summary, we here systematically identified a m6A-related lncRNA signature, which was potently associated with tumor immune microenvironment and immune cell infiltration. Of the six lncRNAs, we found *AC004812.2*, a lncRNA that had not been well characterized, might serve as a regulator of m6A modification and a potential therapeutic target in osteosarcoma. Our results may provide some clues for further investigation, concentrating on the dynamic regulation of lncRNA and m6A modification and its role in oncogenesis and tumor development.

## Data Availability

The original contributions presented in the study are included in the article/Supplementary Material; further inquiries can be directed to the corresponding author.
